# A proposed medical system change in Japan inspired by Swedish primary health care: Important role of general practitioners and specialist nurses at primary health care centers

**DOI:** 10.1002/jgf2.726

**Published:** 2024-09-05

**Authors:** Rie Sato, Ulf Jakobsson, Patrik Midlöv

**Affiliations:** ^1^ Department of Clinical Sciences, Faculty of Medicine, Center for Primary Health Care Research Lund University Malmö Sweden; ^2^ Department of Emergency and Critical Care Medicine, Faculty of Medicine Shimane University Izumo Japan

**Keywords:** general practitioner, medical system change in Japan, primary health care, specialist nurse, Sweden

## Abstract

Japanese citizens of all socioeconomic statuses have benefited from the national insurance system by receiving high‐quality healthcare. However, the Japanese healthcare service is facing a severe financial crisis because of the increasing aging society and social security expenses. Many consultations raise medical expenditure and doctors' work overload, which is about to be regulated, but is questionable how the goal can be achieved without delegating doctors' working tasks. Sweden has a similar health index to that of Japan, but the system is different and is anchored by general practitioners and specialist nurses assigned to primary health care centers. They collaborate to share the workload, responsibilities, and patients' continuous care needs. As a result, the number of consultations is kept small, the length of stay in hospitals is shortened, and doctors' working hours are protected. A system change inspired by Swedish primary health care can be a potential solution for Japanese society.

## Graphical abstract

After graduation from high school, they go to nursing schools and become registered nurses. They take a special education course for specialist nurses after working for at least 1 year as a registered nurse. There are two different special skill education courses for nurses: the specialist nurse program (≥1 year) and the specialization courses.

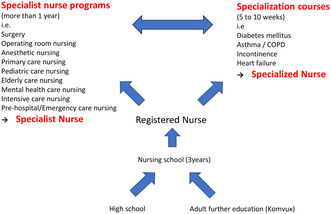

## 1 INTRODUCTION

Japanese healthcare is world‐class in terms of public health and medical results. According to the World Health Report 2000^1^—an index of national health systems' performance—WHO evaluated that Japan was ranked first in the overall health system attainment. In the 2023 ranking of the world's best healthcare systems, Japan was assessed as the second‐best, after Singapore.^2^ However, Japan's aging society^3^ and rising social security expenses,^4^, ^5^ which include pensions, medicine, and welfare, are threatening to collapse the national insurance system due to a financial crisis.^6^ The productive working population, who pay taxes and national medical insurance premiums, is decreasing.^7^ Maintaining the national medical insurance system in this aging society is the most important issue to be addressed in Japan. From a viewpoint of partial structure reforms, *Ikegami* suggested several options that Japan could take.^8^ However, there is a need to change the Japanese medical system drastically.

### 1.1 Japanese medical system and its history

People of all socioeconomic statuses living in Japan have, in principle, benefited from the national insurance system by receiving high‐quality healthcare. Fees and prices are uniform throughout Japan and are, in principle, the same for larger hospitals, such as university hospitals and clinics.^9^ Medical expenditure is paid by the national insurance system, the government, and copayments, which are generally 30% of the whole expenses but are decided according to age or socioeconomic status.^4^ There is also a system called High‐Cost Medical Expense Benefit (Eligibility Certificate for Ceiling‐Amount Application),^10^ which provides patients the privileges to be exempt from payment of a part of the expenses, who received more expensive care than the certain threshold, which is defined according to the municipalities lived and socioeconomic status.

**FIGURE 1:**
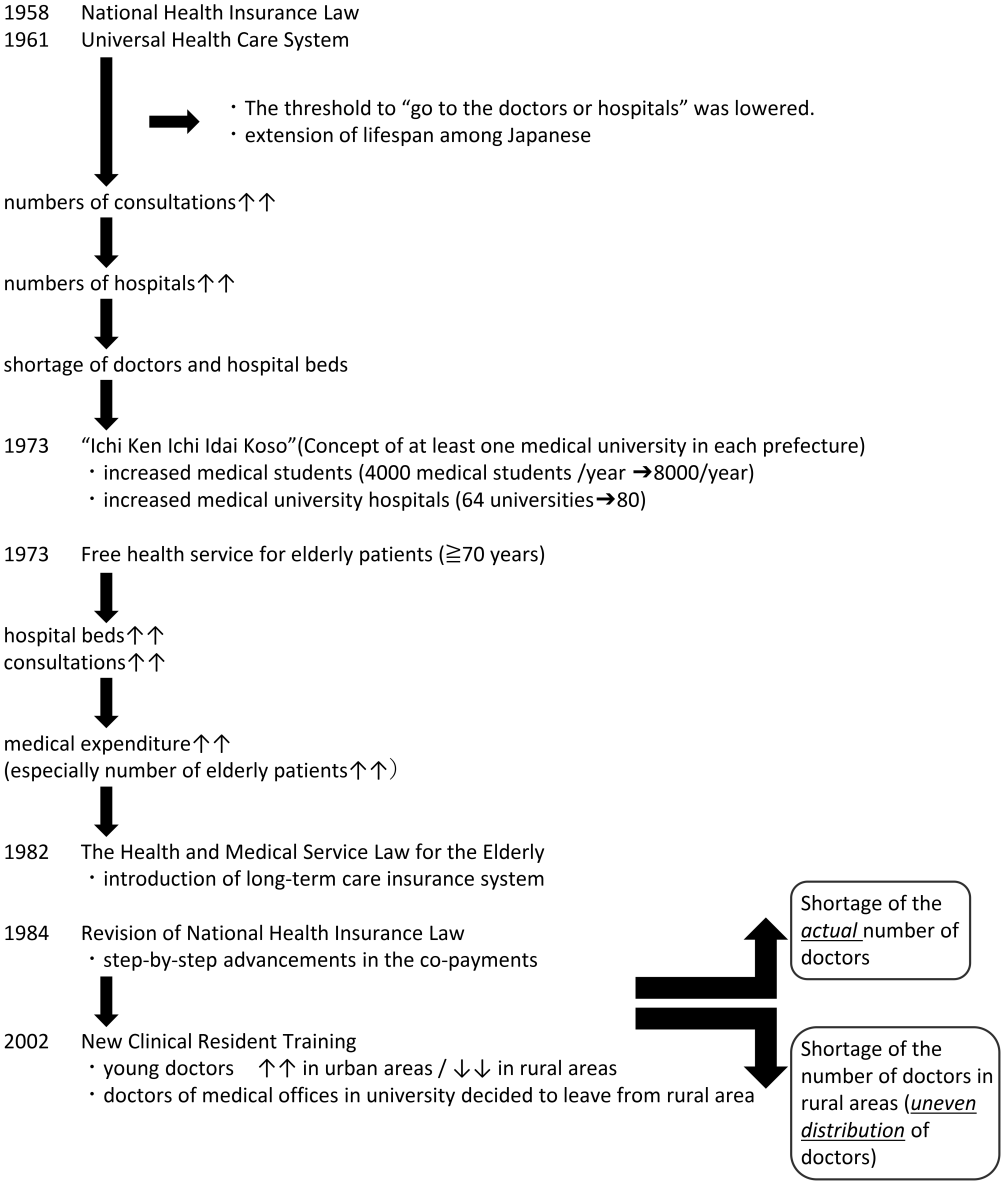
Brief chronological table of the Japanese medical system.

Figure 1 shows a brief overview of the Japanese medical and national insurance system. The Universal Health Insurance System, which was established in 1961, implemented a “free access policy,” enabling Japanese citizens to choose and visit any medical facility, regardless of whether it is public or private, a clinic or a hospital.^11^ The establishment of the Universal Health Insurance System lowered the threshold for Japanese citizens “to go to hospitals and to be seen by doctors,” as the consultation fee was set at a rate that was affordable for everyone. Improvements in nutritional status and sanitation, and declining threshold for consultation contributed to prolonging the lifespan among Japanese citizens.^11^ As more consultations were made, a shortage of doctors and hospital beds emerged. To address these issues, Japan's government decided to make at least one medical university in each prefecture (“*Ichi Ken Ichi Idai Koso*”)^12^ and encouraged hospitals to increase the number of beds. Thanks to this concept, the number of medical schools expanded from 64 (1973) to 80 (1979), and the entire number of graduate students from medical schools nationwide reached 8000/year in 1979, which equated to 150 doctors per 100,000 citizens.^12^ However, *Ichi Ken Ichi Idai Koso* brought more consultations, and medical expenditure was also expanded. The Japanese economy could afford it thanks to the high economic growth it enjoyed at the time, which continued until 1973. The payment system, which was fee‐for‐service, encouraged patients to make more frequent visits and have longer hospitalizations. After the end of the high economic growth period, the Japanese government needed to shrink medical expenditure, but it continued to expand because patients' behaviors, such as frequent visits to multiple medical facilities and long stays in hospitals, had not changed. Health care expenditure, especially for the elderly, increased by 1980.^9^ In 1982, to correct long hospitalization, the Health and Medical Service Law for the Elderly was enacted, in which elderly people, who did not need medical care but could not go home, could change their place to stay from a hospital to elderly care facilities. In 1984, the National Health Insurance Law was revised and the percentage of copayments at the counter in medical facilities has been gradually increasing, including elderly people who have a limited income.

### 1.2 The financial crisis of the Japanese medical system

By 2020, the total medical expenditure of Japan had reached 42.9 trillion yen (300,000 million US dollars), and that of a single Japanese citizen had reached 340 thousand yen (24,000 US dollars).^4^ The average medical expenses of people under the age of 65 was 183,000 yen (approximately 1300 US dollars)^4^ conversely, that of people over the age of 75 was 902,000 yen (approximately 6500 US dollars)^4^; elderly patients need more medical expenditure than younger patients. With the aging society advancing further, the population who need more medical expenses gets larger. The Japanese aging rate (the proportion of those who are 65 years and older) is 28.9%,^3^ which is the highest in the world, and the special total fertility rate is 1.26,^13^ which is one of the lowest numbers. In order to compensate for the budget, the government issued more government bonds, which reached 990 billion yen (approximately 7.1 billion US dollars) in 2021^14^ and the government bond costs per capita have reached 7,130,000 yen (approximately 51,000 US dollars).^14^ As the economy stagnates, incomes have declined.^8^ Workers' premium contribution rates have had to be raised, and more people have not been able to pay premiums.^8^ Adding to this, copayments have been increasing gradually. Although one of the best things about the Japanese national insurance system is that anyone, who lives in Japan, regardless of their social status or income, can receive the same quality of medical examinations, treatments, and medication, these opportunities may be deprived from those who cannot afford premiums and/or copayments. This situation has been an invisible but hard barrier to accessing medicine for the poor, and they have been at risk of being ostracized.

The number of medical consultations in Japan is much higher than in other countries.^15^ The number of doctors' consultations per person was 12.5 in Japan (2019), while the average of the Organization for Economic Co‐operation and Development (OECD) countries is 6.8, which has remained relatively stable since 2009.^15^ The first visit fees or subsequent visit fees are needed for each visit so that more medical expenditure costs are incurred when more consultations are made. According to the historical transition, the fee‐for‐service system is adopted by most clinics and hospitals that mainly have the long‐term care sectors.^8^ In this system, as clinics and hospitals provide more services, they are paid more by the providers, unless they are within the range of the fee schedule. Services, drugs, and devices that are not listed in the fee schedule cannot be provided to clinics or hospitals and they must be paid out‐of‐pocket by clinics or hospitals.^9^ The fee schedule only sets the price, and the healthcare providers are effectively paid according to the fee‐for‐service model.^8^ There are revisions of the fee schedule made in every 2 years that is politically negotiated between the government and providers.^8^, ^9^ The number of hospitals in Japan was approximately 8200 in 2021.^16^ On the other hand, the number of clinics was approximately 104,000.^16^ There are few regulations to open a clinic and a lot of expenditure is paid to many small clinics and hospitals. There is no overviewing system associated with the fee‐for‐service system.^11^ Adding to this, there used to be a prescription rule, in which doctors could not prescribe medication for longer than 90 days in Japan. Although this rule was abolished in 2002,^17^ patients still have this “undesirable custom” of short‐term visits to get prescribed medications, feeling secure by being seen by their doctors frequently, even though their medical conditions are stable. Many hospitals also set their own maximum period for one prescription for their financial management and doctors must follow their policy. As a result of this, almost all doctors encourage patients to visit in less than 90 days.

There are three hypothesized reasons why there are too many consultations in Japan. First, it is reasonable for many patients in Japan to see doctors (the threshold is low for people in Japan to see doctors) thanks to the national insurance system. As the copayment is not expensive, people easily go to see doctors and have consultations even if their health issue is trivial. Second, doctors encourage patients to visit clinics or hospitals frequently, as mentioned above. Third, primary care clinics can receive incentives from the insurance body through the referral,^18^ which encourages more doctors in clinics to refer patients. As many general “physicians” in clinics used to be specialists working at hospitals, they refer their patients to hospitals quite often if the patient's condition is not one of their specialties. If patients received the medical examinations, treatments, and medications at clinics but were referred afterward to hospitals and received similar services, the healthcare bill check and payment organizations reimburse both clinics and hospitals, unless their medical practices are covered by fee schedule.^8^ Each time a patient is seen by a new doctor after a referral, a first visit fee is needed.

Patients' behaviors such as frequent visits and long stays in hospitals, have not changed since 1970, even after the end of high economic growth. Patients visit different specialists with different health problems. Patients also tend to prefer to visit prestigious medical facilities rather than their local clinics.^8^ There are many specialists in hospitals, so they visit hospitals and have multiple consultations. As patients are often referred to hospitals by clinics, lots of patients think it is quicker to visit hospitals directly by themselves without needing a referral. To reduce patients' visits to larger hospitals, they set an extra payment for patients without a referral^8^; however, in general, most patients do not care about fees. Japanese patients tend to be hospitalized for a longer time compared to other countries.^19^ The average length of a hospital stay in 2019 was 16.0 days in Japan,^19^ while the average in OECD 38 countries was 7.6 days.^19^ This is kind of remnant of historical long hospitalization before The Health and Medical Service Law for the Elderly. There are some reasons why doctors cannot discharge patients without concern. Many doctors cannot persuade patients or patients' families to be discharged early and be cared for at home. Japanese patients and their families continue to desire to remain longer in the hospital for their safety and “peace of mind,” as they can “feel secure” when the patients are hospitalized, by being closer to the health care providers and facilities. At the same time, there is a shortage of primary care doctors who have completed their special education for primary care all over the country, as a new training program for primary care doctors was launched in Japan in 2018.^20^, ^21^ In this situation, doctors working in hospitals sometimes cannot find suitable primary care doctors in the community, which could be the reason for longer stays in hospitals.

### 1.3 Doctors' overload as a result of the current medical system in Japan

In general, Japanese doctors being overworked is a major problem.

Historically, Japan widely adopted German medical practices in the mid‐1800s,^9^, ^22^ which respect research‐oriented medicine, not clinical pathology‐oriented medicine. In Japan, every medical university has its own university hospital and several medical offices (*Ikyoku*).^22^ The *Ikyoku* system originated in Germany.^22^, ^23^ *Ikyoku* has three primary goals: education, research, and patient care.^22^ *Ikyoku* provides young doctors with specialized education, so many young doctors tend to be willing to be in *Ikyoku* and become specialists. Nowadays, most doctors have been expected to be specialists by both doctors and citizens, and many young doctors tend to want to become specialists and they start specialist training just after they complete their primary resident training. Not only doctors but also Japanese society have accepted the concept of specialists‐centered medicine in this historical stream. Many patients tend to care about the doctors' specialization, and they are eager to be seen by each specialist even if their health problems are not serious or highly specialized enough to concern specialists. In university hospitals, many consultations not only keep doctors at the outpatient clinic for a longer time but also other important roles such as researchers and educators are difficult to fill. Doctors working at hospitals must be responsible for inpatient management. The workload of those in middle to small public hospitals in rural areas, where there is an extreme shortage in the number of doctors, is serious. As mentioned previously, *Ikyoku* is symbolic of a Japanese university hospital,^22^ which has a strong enough influence to decide the distribution of doctors in each area. The *Ikyoku* group has sent doctors to affiliated hospitals based on political and financial reasons,^22^ but they meet the requirements of each hospital's demands with the placement of doctors.^19^ In Japan, most of the resident doctors, who graduated from medical universities, complete their first 2‐year training period in hospitals (primary resident training) as the basis for two Japanese acts: the Medical Practitioners' Act^24^ and the Medical Care Act.^25^ Medical Practitioners' Act stipulates that a medical practitioner, who graduated from medical universities, and “who seeks to engage in medical practice must undergo clinical training for a period of 2 years or more at a university hospital with a medical science course or at a hospital designated by the Minister of Health, Labour and Welfare”. The Medical Care Act also stipulates finishing their resident training to become a hospital or clinic administrator. After the initiation of a primary resident system for doctors in 2002, trainees can choose the hospital they are trained in freely from all over Japan (Figure 1). Before that system started, young doctors tended to stay in university hospitals from which they graduated, and they tended to enter the *Ikyoku* straight after finishing medical universities.^22^, ^26^, ^27^ After the new system was initiated, however, young doctors tended not to stay in their alma mater and started working in urban areas,^8^ with not many young doctors staying in rural areas. After the outflow of young doctors to cities, the shortage of resources in *Ikyoku* in universities in rural regions also became severe, and they became reluctant to send support doctors to middle‐ and small‐scale public hospitals in rural areas, which made the situation more serious. Doctors in middle‐ to small‐sized public hospitals in rural areas suffered due to the heavy workload of many outpatient consultations and inpatient management, as a result of the shortage of human resources, including not only the decrease of full‐time workers but also that of part‐time workers who used to be sent by *Ikyoku*. This uneven distribution of doctors all over Japan makes doctors who are working in those hospitals exhausted because of heavy workloads. Some doctors gave up practicing medicine due to the hard working conditions and huge responsibilities in the hospital and opened their own clinics. The total number of doctors working in such hospitals is decreasing even further and, as a result, their workloads are getting even greater. This phenomenon could have been causing the collapse of community medicine in rural areas.

Maximum working hours for all occupations is set up to 40 h per week by law in Japan (enacted in 1947).^28^ However, lots of doctors have been working extra hours as “exceptions”—working overtime, on holidays, day duty, and night duty is admitted. In fact, there is currently no ceiling time for doctors' working hours in Japan. The Ministry of Health Labor and Welfare announced the reality of doctors' working hours in 2006, which revealed that Japanese‐employed doctors worked, on average, 79.6 h per week.^29^ To improve this situation, The Ministry of Health, Labor and Welfare of Japan is launching “Work Style Reform”.^30^ From 2024, the system with no ceiling time for doctors' working hours will be abolished, and all doctors must finish their work within the limitation of the working time. If they work for longer than the limitation, the institution hiring the doctors will incur penalties. However, it is questionable how this goal can be achieved without delegating doctors' duties and working tasks. Japan has started to accept nurses from other countries, but they must have first mastered the Japanese language in order to practice medicine in Japan. It is also difficult for foreign doctors to work in Japan as they have to pass the Japanese national examination to practice medicine in Japan.

### 1.4 Comparison between Japan and Sweden

Sweden is referred to as a country that has one of the most sophisticated social health systems.^31^ Some of the social and health indexes are similar between Japan and Sweden. According to OECD and the World Bank, there are many similarities between the two countries such as the fertility rate [Japan 1.30 (2021)/Sweden 1.67 (2021)],^32^ the health expenditure as a share of GDP [both Japan 10.90% (2020)/Sweden 11.28% (2021)],^33^ the health spending per capita [Japan $4388.10 (2020)/Sweden $6914.91 (2021)],^34^ the life expectancy at birth [Japan 84.5 (2021)/Sweden 83.1 (2021)],^35^ the infant mortality rate (Japan 1.7/1000/Sweden 1.8/1000),^36^ the children vaccination rates [Measles; Japan 95.4% (2021)/Sweden 92.5% (2022), diphtheria, tetanus, pertussis; Japan 98.8% (2021)/Sweden 94.3%(2022)].^37^

The financial system concerning medicine is also similar. The Japanese medical system and the nursing and welfare service system are controlled by the Ministry of Health, Labor, and Welfare, which is at a national level. About 40% of the health care expenditure is funded by taxation, 50% is funded by insurance fees, and the rest is funded by copayments.^4^ On the other hand, Sweden's health care system is organized on three levels: national, regional, and municipal level. At the national level, the Ministry of Health and Social Affairs has a role in establishing the policies. There are 21 counties (also known as regions) in Sweden and those counties have the responsibility to provide medicine and health care, such as specialist care at hospitals and primary health care at Primary Health Care Centers (PHCCs), at the regional level. The municipalities are responsible for home care at the municipal level, providing nursing and welfare services. The medical system in Sweden is funded by taxation. About 70% of the health care expenditure was funded through local taxation and 17% was supported by general state grants in 2009.^38^

Table 1 shows the comparison of some figures between Japan and Sweden.^15^, ^19^, ^39^, ^40^, ^41^, ^42^, ^43^, ^44^ There are three features: First, there is a shortage of doctors in Japan compared to Sweden. Second, there are too many consultations in Japan compared to Sweden. Third, Japan has an excessive number of hospital beds compared to Sweden.

**TABLE 1:** Comparison of main health indicators in Japan and in Sweden (the OECD Data).

	Sweden	Japan
Number of practicing doctors per 1000 inhabitants^34^	4.3 (2020)	2.6 (2020)
Number of nurses per 1000 inhabitants^35^	10.7 (2020)	12.1 (2020)
Number of Doctors' consultations per person^a^ ^,^ ^15^	2.6 (2019)	12.5 (2019)
Estimated number of consultations per doctor^15^	625 (2019)	5011 (2019)
Hospital beds	21,288 (2019)^36^	1,500,600 (2021)^37^
Hospital beds per 1000 inhabitants^38^	2.0 (2021)	12.6 (2021)
Length of hospital stay in hospital for all causes^18^	5.6 (2019)	16.0 (2019)
Hospital discharge rates per 100,000 populations^b^ ^,^ ^39^	13,012 (2021)	12,327 (2020)

^a^ Doctors' consultations per person: the number of consultations patients have with doctors in a given year.

^b^ Hospital discharge rates: the number of patients who leave a hospital after receiving care. Hospital discharge is defined as the release of a patient who has stayed at least one night in hospital. It includes deaths in hospital following inpatient care.

Sweden has similar health index outcomes to Japan. However, it has a totally different medical system from that in Japan. To address those issues mentioned above, the aim of this article is to introduce the concept of the Swedish GP‐centered medical system and various specialist nurses into Japanese society.

## 2 SWEDISH MEDICAL SYSTEM

### 2.1 General practitioner‐centered primary care system and cooperation with specialists in hospitals

The Swedish primary health care system is anchored by the general practitioners (GPs) and specialist nurses. All residents of Sweden are on the list of their specific primary health care center (PHCC) located in their residential area. GPs are responsible for the patients who are assigned to their PHCC lists, having trained to cover not only physical but also mental problems. For example, GPs can manage to cover the patient's hypertension, diabetes mellitus, and osteoarthritis in their 20‐min consultation time. Patients do not need to go to different outpatient clinics to be seen by multiple specialists, such as a cardiologists, metabolic physicians, or orthopedic physicians, like in Japan. GPs follow their patients for a long time and patients' information is transferred to the new GP if the patients change their PHCC. GPs are responsible for taking telephone consultations from patients directly, which are scheduled by nurses, according to the requests of the patients. There is no charge for the telephone consultation fee. These systems are made possible due to the continuous care of the same patient by the same GPs. The strong trust relationship between the patient and the GP enables the maintenance of this reasonable practice style all over Sweden. Thanks to those systems, the number of consultations is suppressed, which brings a reduction in medical expenditure.

In Sweden, the average working hours are 40 h per week, because of the Working Hours Act,^45^ which states that regular working time may not exceed 40 h per week. Doctors are no exception. The reason for the suppression of doctors' working hours is the contribution of cooperation between GPs and specialists, and the collaboration of well‐trained GPs and specialist nurses. GPs working in PHCCs play a major role in taking care of patients with common diseases, which include both acute and chronic diseases, in the outpatient clinic. Doctors working in hospitals, who are primarily specialists, can concentrate on patients' special care or treatments, so that they do not have to spend a long time in outpatient clinics with common diseases. Specialists working at hospitals are not exhausted by their many outpatient consultations, and GPs can concentrate on outpatient clinics, and not be disturbed by inpatient management. After the completion of special care at hospitals, specialists refer back to the GPs for their continuous care without any concerns.

It is GPs' right to adjust their working hours. It is quite possible for them to have shorter working hours because of the cooperation of other GPs working at the same PHCC; GPs are covering other GPs' shortage of working hours, which makes it possible for young GPs to have pregnancies or child‐births, and child‐raising, and to study at postgraduate schools to get a PhD. For example, if a GP is studying for a PhD, he or she can reduce their working hours to 80%, and the rest of the time they can use for studies. If a GP has a small child, he or she can reduce his or her working hours to 50%, and the rest of the time they can spend with his or her family. This percentage decrease affects their salary.

In summation, the GP‐centered medical system may bring a lot of benefits to patients, society, and to the doctors themselves.

### 2.2 Important role of specialist nurse

#### 2.2.1 Specialist nurses are in charge of independent consultations

There are specialist nurses in Sweden, who are independently responsible for taking care of patients without a doctor's observation. Thanks to them, doctors can concentrate on their patients who need more urgent or specific care. Moreover, the copayment is free when a patient is seen by a specialist nurse, even with treatment or prescription. In Sweden, the consultation frquency by doctors is very limited, compared to Japan. It is quite normal for Swedish patients with chronic disease to visit their GP twice or even just once a year if their conditions are stable. Specialist nurses play a great role in following up those patients. For example, patients with diabetes mellitus (DM) visit their doctor once a year, as they are seen by a diabetic nurse, which is one of the specializations. If a patient's condition is stable, diabetic nurses can order insulin, including changing the doses of insulin. If there is any change in patients' condition, specialist nurses consult their GP and make an appointment. However, only doctors can prescribe diabetic medications. The situation for patients with asthma is similar. Patients are seen by asthma/chronic obstructive pulmonary disease (COPD) nurses regularly, even once every 2–4 years if their medical conditions are stable, i.e., without any deterioration. At every PHCC, there must be at least one diabetes nurse and an asthma/COPD nurse. They see specific patients, but they also do what registered nurses do in the PHCC.

Specialist nurses have specific specialized knowledge and skills, and play great roles in daily practice, especially in primary care settings (i.e., district nurses). These specialist nurses have their own examination rooms and run their own outpatient clinics.

#### 2.2.2 The education of specialist nurses

**FIGURE 2:**
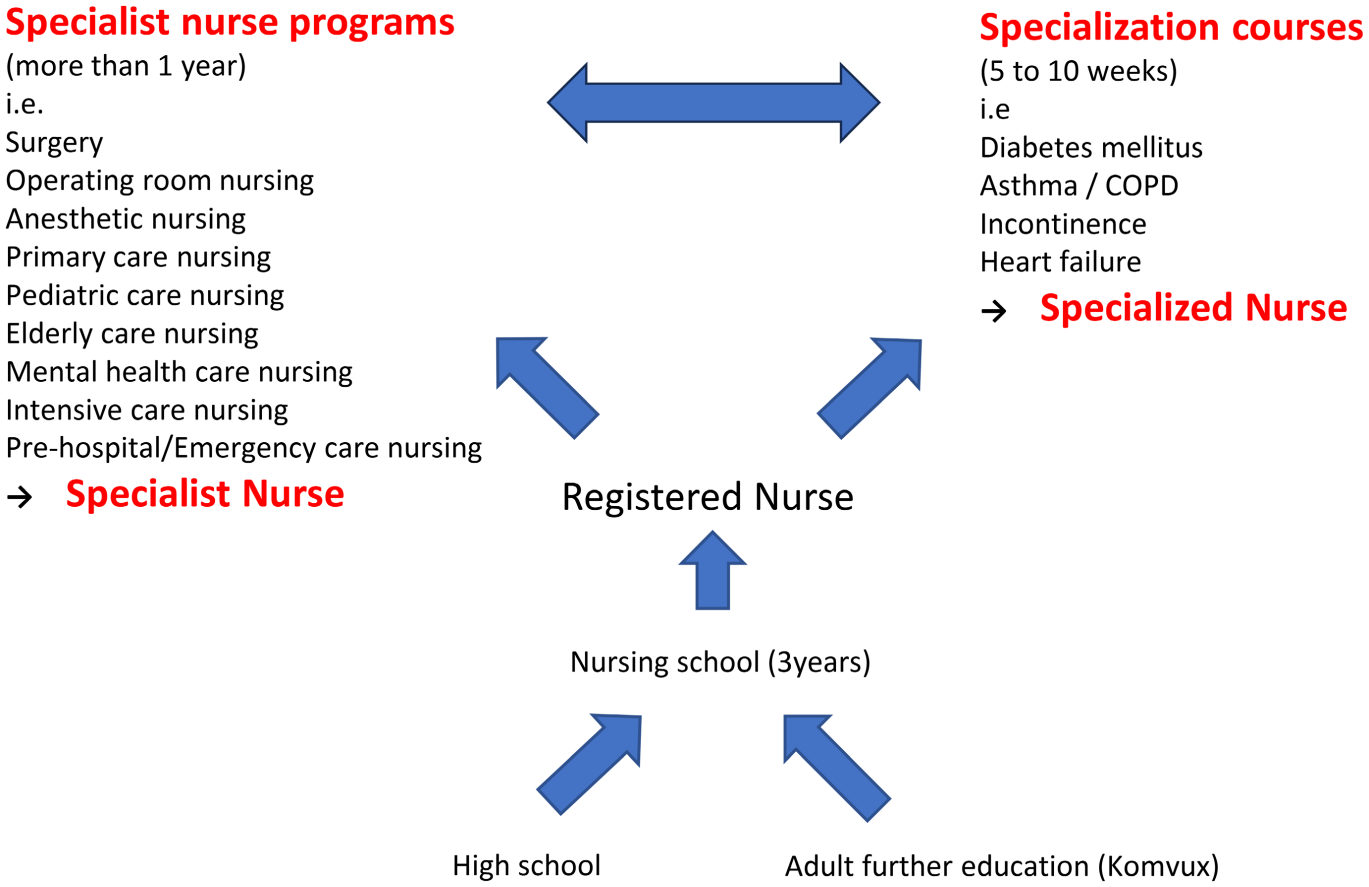
The pathway to become specialized nurses.

Figure 2 shows the process of how to become a specialist nurse and a nurse completed specialization. After graduation from high school, they go to nursing schools (universities) for 3 years, get a diploma (nursing degree), and become registered nurses. If they want to become specialist nurses, they take a special education course for specialist nurses after working for at least 1 year as a registered nurse. There are two different special skill education courses for nurses: the specialist nurse program (≥1 year) and the specialization courses. The specialist course offers education for, e.g., surgery, operating room nursing, anesthetic nursing, primary care, pediatric care, elderly care, mental health care, intensive care, prehospital, and emergency care. The specialization courses are shorter (around 5–10 weeks focusing on a specific topic) and offer education for, e.g., diabetes mellitus nurses, asthma/COPD nurses, incontinence nurses, and heart failure nurses. The educational duration of the time for the specialist course differs with the specialties, but it usually takes about a year to complete. For example, it takes 1 year to become an anesthesia nurse, surgery nurse, child health care nurse, geriatrics (elderly care) nurse, and intensive care nurse, but almost 1.5 years to become a district nurse who works at a PHCC. District nursing education is a specialist area at the postgraduate level to deepen nurses' knowledge, skills, and capacity by building on undergraduate education, and eventually developing the independence to deal with complex phenomena, issues, and situations.^46^ Only district nurses have the authorization to prescribe medications included in their education (i.e., complementary training in pharmacology at an advanced level). District nurses are authorized to work in several different areas (e.g., primary health care, child health care, community care, and school nursing), in which they usually work more independently than other specialist nurses, so they need a longer training period. They often need to make a diagnosis and start treatment immediately on their own at the primary care sites, hence they need not only therapeutic knowledge and skills but also diagnostic training.

#### 2.2.3 District nurses

A specialist nurse in Swedish primary health care is called a district nurse (There are several specialized nurses working in primary health care in Sweden). At the primary care site, district nurses play very important roles. They cooperate with GPs and they organize health care teams. In Sweden, district nurses are well‐trained health professionals providing essential services, who are not posing as doctor substitutes.^47^ They are responsible for several duties. First, all district nurses can prescribe certain kinds of medication and order vaccines. The authorization to prescribe medicine is limited but covers a wide range of medications, for example, analgesics, antifungals, cortisone creams, estrogen, and some antibiotics. Tetanus, hepatitis, pneumococcal, influenza, and COVID‐19 vaccination are examples of vaccines that district nurses can order without a doctor's permission. Second, district nurses are responsible for triage of the patients at the outpatient clinic and the telephone consultations. Their judgment is very important as they could change the patients' prognoses. If a patient visits a PHCC without an appointment, the first contact should be taken by district nurses. They make an assessment of whether the patient needs urgent treatment, then refer to the GP in charge of the day, and start initial treatment at the same time if needed. If they don't think that immediate treatment is needed, they make an appointment on the doctor's schedule and send them home. Correct and clear instructions during telephone consultations are also essential. A judgment on whether the patient should visit a doctor immediately, observe for a certain period, or not need to visit a doctor must be made. Thus, district nurses need well‐trained skills, broad knowledge, and enough experience to assess the patient's condition appropriately based on limited information. They play an important role as gatekeepers to avoid unnecessary patient visits to PHCCs. There are guidelines for medical triage and how nurses should deal with various situations firsthand. Third, district nurses also have their own reception taking care of patients' wounds and dermatological treatments (eczema, mycosis infections, etc.) without instructions or observations from a supervising doctor. They can diagnose, prescribe medicine, arrange the treatment plans, make appointments, and follow up until the wound/skin condition heals. Fourth, district nurses can also work in child health care or school nursing (including regular child checkups and immunizations). Child health care nurses can also work in this fourth area. Fifth, district nurses can visit a patient's house and give some care with or without the presence of a doctor. At home care, district nurses visit patients individually, and they often encounter complex conditions. District nurses in PHCCs play a key role in health‐promotive/disease‐preventive work, meeting people suffering from and/or having risks for noncommunicable diseases, such as poor diet, smoking, and alcohol use.^48^

## 3 DISCUSSION

Swedish primary health care is anchored on GPs, PHCCs, and specialist nurses. The cooperation of GPs and specialists, and the collaboration of GPs and specialist nurses ensures patients' continuous care and a cost‐efficient system. A system change inspired by Swedish primary health care will be beneficial for Japanese society in several ways.

### 3.1 The establishment of the medical system, centering on GPs and PHCCs

The establishment of the medical system centered on GPs and PHCCs is hypothesized to potentially bring several merits to patients, Japanese society, and doctors.

In the GP‐ and PHCC‐centered medical system, patients do not have to go to multiple different clinics and/or hospitals—they just visit their GP. When patients are hospitalized, they could be treated by specialists at the hospital but soon after they get better, they are discharged home as GPs can take care of them there. There is no major risk of overlapping prescriptions.

Regarding medical expenditure, first‐visit fees and subsequent visit fees could be cut, as the number of consultations would be reduced. As mentioned previously, the Japanese medical society adopted German medical practices, which is also called scientific medicine, emphasizing investigation of etiological agents and research, not English medical practice, which is also called practical medicine, emphasizing prevention and treatment.^49^ Therefore, Japanese medical practices and education have tended to be German style in the present—research centered medicine—not practice centered medicine, of which GPs are one of the symbols. In Japan, the concept of “general physicians” has a quite long history. Most doctors who currently take care of patients at their local clinics are those who used to be specialists and have not received specific training for GPs. They actually have been helping people with various health problems for a long time. The specialist system was launched about 50 years ago, and since then, each relevant professional association has provided special education, training, and approval of medical specialists. After being active in specialist fields, some doctors, whose family own their private clinics, or those who want or need to change their lifestyle, quit working in hospitals and start running their own clinics. Those doctors have been playing the role of “general physicians” in Japanese society. In 1968, The Japanese Society of Internal Medicine started to approve general physicians.^50^ However, the special education for “general practitioners” (GPs) was not established systematically. In 2018, the Japanese Medical Specialty Board approved general practice as a specialty and launched systematic education and training programs for GPs.^20^, ^21^ However, there are not many GPs who have completed their special GP training in Japan thus far. If GPs can manage the physical and mental problems of patients widely and refer to hospitals appropriately, duplicated medical expenditure, which includes medical examinations, treatments and medications, could be cut. If PHCCs are arranged according to the number of residents, the excessive number of clinics could be reviewed and medical expenditure would be cut with a decrease in clinics, in which medical fees are set according to fee‐for‐service.

Doctors could also benefit from this system. As mentioned previously, Japanese doctors, especially those working at hospitals, must spend longer hours in charge of outpatient clinics, adding to inpatient management. If GPs are properly in charge of patient care after discharge, specialists working at the hospital can send patients home quickly without any concern. Patients can go home and their families can receive them without big concerns, then long hospitalizations would decrease. When doctors get tired, their work efficiency gets worse, and more tasks and work lie on their shoulders. As doctors' performance status gets worse, it becomes difficult for them to provide appropriate patient‐specific medical examinations and therapies, thus leading to more unnecessary medical examinations and treatments taking place, which brings a greater financial burden. And, of course, patient safety—the most important issue in medicine—can be in danger. As a result of the aging society, doctors' duties tend to keep increasing, especially the management of elderly patients who have many different health and social problems at the same time, and whose situations are very complicated. If GPs and specialists, who are working in hospitals, could share the responsibilities, neither of them would have to be burdened with outpatients and/or inpatients who are staying for longer periods of time.

However, there are some known obstacles that need to be handled when implementing a new proposed primary health care system in Japan. As the new training system for GPs for young doctors has already been launched in Japan, fortunately, society and medical education can encourage more young doctors and medical students to become GPs. The most important thing to ensure a GP‐ and PHCC‐centered medical system is thought to be the “preparation of the society”; it would be essential to establish the environment in which the Japanese society fully accepts it. Without it, GPs cannot play important roles in society nor be given enough responsibilities in the primary care site based on their “special training” and professionalism. It is very important to educate citizens to “trust” their GPs and to encourage them to visit their GPs in primary care settings. The reconstruction of clinics will be essential, in which public medical centers, such as PHCCs, would be located according to the city scales and populations.

### 3.2 Introduction of “specialist nurses” to Japan

There is no medical profession that exists in Japan like Swedish specialist nurses. If Japanese society implemented a specialist nurse system, at least three ideal changes would be expected to happen. First, specialist nurses could share the workload with doctors, and the number of consultations for each doctor would decrease. In Sweden, the number of patients who visit the PHCC is controlled thanks to district nurses' appropriate triages. Doctors could be free from the burden and responsibilities of excessive patients, and they could have a longer period of time for each consultation so that patients would be more satisfied. Adding to this, a specialist nurse system could be the solution for “Working Style Reform” in Japan. Concerning the excessive working hours among doctors, the Japanese government has decided to abolish the system with no ceiling time for doctors' working hours in 2024, as mentioned previously. To address this issue, specialist nurses could be the ones who can be able to cover doctors' medical practice since they can practice both in collaboration with GPs and independently. Second, if the fees of consultations for specialist nurses are set differently, a lot of medical expenditure can be reduced. If the copayment with specialist nurses' consultations is set to be free as in Sweden, the adoption of specialist nurses in society would be accelerated. This is also beneficial to keep following the poor as they can keep visiting their specialist nurses without worrying about the payments. Third, specialist nurses would be able to feel a sense of achievement and be satisfied, and they would be encouraged to improve their knowledge and skills more for the patients because they could have greater workplace privileges and society would regard them as independent decision‐makers. There are many excellent Japanese nurses who are, unfortunately, not fully contributing to the Japanese medical society because of the limitation of their workplace privileges. It is a worthwhile idea to introduce the specialist nurse system to Japan to share the responsibilities among doctors and specialist nurses. Many highly qualified nurses quit their professions and never come back to practice due to pregnancies and childcare responsibilities in Japan. If nurses' work could be much more attractive, the number of nurses who return to their profession would increase. This could be a solution to the shortage of human resources in nursing sites, which is another serious problem all over Japan.

## 4 CONCLUSION

Japan's healthcare service has faced a severe financial crisis with the aging society, inefficient finances, and undesirable patient behavior, such as many consultations, longer stays in hospitals, which make Japanese doctors exhausted by outpatient and inpatient management. A wide introduction of a GP‐ and PHCC‐centered medical system to Japanese medical society would bring various merits to patients, doctors, and society. It is a new but important idea to share the workload and responsibilities of patients with multiple medical problems with various medical specialties, such as GPs, specialists, and specialist nurses. It can be one of the solutions for the financial crisis that the Japanese healthcare service is now facing, and the doctors' overwork, namely, “Working Style Reform.”

If doctors' workload burden and medical expenditure continue in tandem with the severely aging society, the collapse of the national insurance system is thought to be waiting for Japan without taking any action. It is hypothesized that the introduction of a Swedish medical system could be a potential savior of Japanese society.

## CONFLICT OF INTEREST STATEMENT

This study has been partly supported by the International Dispatch Program 2019 from the Japan International Cultural Exchange Foundation and Overseas research fellowships from the Uehara Memorial Foundation (No 20200507). The foundations had no role in this study including design data collection and analysis, interpretation of results, the decision to publish, or preparation of the manuscript.

## References

[1] World Health Organization . World Health Report 2000. Health systems: improving performance.

[2] International citizens insurance, ranking the best healthcare in the world by country. https://www.internationalinsurance.com/health/systems/ [cited on 6th December, 2023]

[3] Cabinet Office, Japan . Annual report on the aging society in 2022. https://www8.cao.go.jp/kourei/whitepaper/w‐2022/html/zenbun/s1_1_1.html [in Japanese] [cited on 6th December 2023]

[4] The Ministry of Health, Labor and Welfare, Japan . The overview of national medical expenses in 2020. https://www.mhlw.go.jp/toukei/saikin/hw/k‐iryohi/20/dl/data.pdf [in Japanese] [cited on 6th December, 2023]

[5] The Ministry of Health, Labor and Welfare, Japan . Benefits and charge in social security expenses. https://www.mhlw.go.jp/stf/newpage_21509.html [in Japanese] [cited on 6th December, 2023]

[6] Cabinet Office . The trend of the medical expenditure. 2016. https://www5.cao.go.jp/keizai‐shimon/kaigi/special/reform/wg1/280711/shiryou3.pdf [in Japanese] [cited on 6th December, 2023]

[7] The Ministry of Internal Affairs and Communications . Information and communication white paper. 2022. https://www.soumu.go.jp/johotsusintokei/whitepaper/ja/r04/html/nd121110.html [in Japanese] [cited on 6th December, 2023]

[8] Ikegami N , Campbell JC . Japan's health care system: containing costs and attempting reform. Health Aff. 2004;23(3):26–36. 10.1377/hlthaff.23.3.26 15160800

[9] Ikegami N , Campbell JC . Dealing with the medical axis‐of‐power: the case of Japan. Health Econ Policy Law. 2008;3:107–113. 10.1017/S1744133108004428 18634623

[10] The Ministry of Health, Labor and Welfare, Japan . High‐cost medical expense benefit. https://www.mhlw.go.jp/content/000333279.pdf [in Japanese] [cited on 6th December, 2023]

[11] Hirose M , Imanaka Y , Evans E , Ishizaki T . How can we improve the quality of health care in Japan? Learning from JCQHC hospital accreditation. Health Policy. 2003;66:29–49. 14499164 10.1016/s0168-8510(03)00043-5

[12] The Ministry of Health, Labor and Welfare, Japan . Discussion committee of demand of health care workers. The historical background of demand and supply of doctors. https://www.mhlw.go.jp/file/05‐Shingikai‐10801000‐Iseikyoku‐Soumuka/0000199257.pdf [in Japanese] [cited on 6th December, 2023]

[13] The Ministry of Health, Labor and Welfare, Japan . Total fertility rate in 2022. https://www.mhlw.go.jp/toukei/saikin/hw/jinkou/geppo/nengai22/dl/tfr.pdf [in Japanese] [cited on 6th December, 2023]

[14] Ministry of Finance, Japan . The national plan for issuance of government bonds in 2021. https://www.mof.go.jp/jgbs/publication/debt_management_report/2021/saimu2021‐1‐2.pdf [in Japanese] [cited on 6th December, 2023]

[15] OECDiLibrary . Health at a glance 2021: OECD indicators. Number of in‐person doctor consultations per person. Estimated Number of in‐Person Consultation per Doctor. https://www.oecd‐ilibrary.org/sites/b088de1d‐en/index.html?itemId=/content/component/b088de1d‐en [cited on 6th December, 2023]

[16] The Ministry of Health, Labor and Welfare, Japan . The report of medical facilities investigation in 2021. https://www.mhlw.go.jp/toukei/saikin/hw/iryosd/21/dl/02sisetu03.pdf [in Japanese] [cited on 6th December, 2023]

[17] The Ministry of Health, Labor and Welfare, Japan . A revision of the medical law for medical safety measures. https://www.mhlw.go.jp/topics/bukyoku/isei/i‐anzen/2/kaisei/index.html [in Japanese] [cited on 6th December, 2023]

[18] Kato D , Ryu H , Matsumoto T , Abe K , Kaneko M , Ko M , et al. Building primary care in Japan: literature review. J Gen Fam Med. 2019;20:170–179. 10.1002/jgf2.252 31516802 PMC6732569

[19] OECDiLibrary . Health at a glance 2021: OECD indicators. Hospital discharges and average length of stay. https://www.oecd‐ilibrary.org/sites/18faaea9‐en/index.html?itemId=/content/component/18faaea9‐en#figure‐d1e5273 [cited on 6th December, 2023]

[20] Japanese Medical Specialty Board . https://jmsb.or.jp/ [in Japanese] [cited on 6th December, 2023]

[21] Watari T , Hirose M , Midlöv P , Tokuda Y , Kanda H , Okayama M , et al. Primary care doctor fostering and clinical research training in Sweden: implications for Japan. J Gen Fam Med. 2019;20:4–8. 10.1002/jgf2.211 30631652 PMC6321823

[22] Kuwabara N , Yamashita M , Yee K , Kurahara D . The evolution of the Japanese medical education system: a history perspective. Hawaii J Med Public Health. 2015;74(3):96–100. 25821652 PMC4363931

[23] The Ikyoku System . The Hokkaido News Paper. Published May 17, 2003.

[24] Minister of Health, Labour and Welfare, Japan . Medical Practitioners' Act. https://www.japaneselawtranslation.go.jp/en/laws/view/3992 [cited on 13th July, 2024]

[25] Minister of Health, Labour and Welfare, Japan . Medical Care Act. https://www.japaneselawtranslation.go.jp/en/laws/view/4006 [cited on 13th July, 2024]

[26] Otaki J . Considering primary care in Japan. Acad Med. 1998;73:662–668. 9653405 10.1097/00001888-199806000-00013

[27] Yoshida A . What is the problem in Japanese medicine. Tokyo: NTT Publishing Co.Ltd; 2009. p. 320.

[28] The Ministry of Health, Labor and Welfare, Japan . Labor Standards Act. https://www.mhlw.go.jp/web/t_doc?dataId=73022000&dataType=0&pageNo=1 [in Japanese] [cited on 6th December, 2023]

[29] The Ministry of Health, Labor and Welfare, Japan . Masahiko Hasegawa. The current status of labor environment of doctors. https://www.mhlw.go.jp/shingi/2006/03/s0327‐2d.html [in Japanese] [cited on 6th December, 2023]

[30] The Ministry of Health, Labor and Welfare, Japan . Work style reform. https://hatarakikatakaikaku.mhlw.go.jp/ [in Japanese] [cited on 6th December, 2023]

[31] Watari T , Hirose M , Midlöv P , Okayama M , Yoshikawa H , Onigata K , et al. Japan can learn from the Swedish primary care doctor fostering system. J Gen Fam Med. 2018;19:183–184. 10.1002/jgf2.197 30186735 PMC6119798

[32] OECD Data . Fertility rates. https://data.oecd.org/pop/fertility‐rates.htm [cited on 6th December, 2023]

[33] The World Bank . Current health expenditure (% of GDP). https://data.worldbank.org/indicator/SH.XPD.CHEX.GD.ZS [cited on 6th December, 2023]

[34] The World Bank . Current health expenditure per capita (current US$). https://data.worldbank.org/indicator/SH.XPD.CHEX.PC.CD [cited on 6th December, 2023]

[35] OECD Data . Life expectancy at birth. https://data.oecd.org/healthstat/life‐expectancy‐at‐birth.htm [cited on 6th December, 2023]

[36] OECD Data . Infant mortality rates. https://data.oecd.org/healthstat/infant‐mortality‐rates.htm [cited on 6th December, 2023]

[37] OECD Data . Child vaccination rates. https://data.oecd.org/healthcare/child‐vaccination‐rates.htm [cited on 6th December, 2023]

[38] WHO Health Systems . WHO health systems in transition, Sweden health system review. 2012, Vol.14 No 5. https://apps.who.int/iris/handle/10665/330318 [cited on 6th December, 2023]

[39] OECD Data . The number of doctors, per 1000 inhabitants. https://data.oecd.org/healthres/doctors.htm [cited on 6th December, 2023]

[40] OECD Data . The number of nurses, per 1000 inhabitants. https://data.oecd.org/healthres/nurses.htm [cited on 6th December, 2023]

[41] Statista . Number of hospital beds in Sweden from 2000 to 2019. https://www.statista.com/statistics/557358/hospital‐beds‐in‐sweden/ [cited on 6th December, 2023]

[42] Statista . Number of beds available in hospitals in Japan from 2012 to 2021. https://www.statista.com/statistics/624151/japan‐number‐hospital‐beds/ [cited on 6th December, 2023]

[43] OECD Data . Total hospital beds, per 1 000 inhabitants. https://data.oecd.org/healtheqt/hospital‐beds.htm [cited on 6th December, 2023]

[44] OECD Data . Total hospital discharge rates, per 100 000 inhabitants. https://data.oecd.org/healthcare/hospital‐discharge‐rates.htm [cited on 6th December, 2023]

[45] Sweden Government . Working Hours Act. https://www.government.se/contentassets/1b29fd35b2544f13875137beab80911a/1982673‐working‐hours‐act.pdf [cited on 6th December, 2023]

[46] Jarnulf T , Skytt B , Mårtensson G , Engström M . District nurses experience of precepting district students at the postgraduate level. Nurse Educ Pract. 2019;37:75–80. 10.1016/j.nepr.2019.05.004 31128519

[47] Ejlertsson G , Jansson AK . The district nurse and the district physician in health care teams. An analysis of the content of primary health care. Scand J Prim Health Care. 1987;5:73–78. 3616276 10.3109/02813438709013980

[48] Linden GF , Jacobsson S , Lidén E , Björkman I . District nurses' perspectives on health‐promotive and disease‐preventive work at primary health care centres: a qualitative study. Scand J Caring Sci. 2023;37(1):153–162. 10.1111/scs.13100 35778918

[49] Nakayama K . ドイツ医学とイギリス医学の対立が生んだ森田療法‐森田理論をその源流から探る. Psychiat Neurol Jap. 2008;110(8):698–705. [in Japanese].

[50] Watabane T . The role of internal medicine specialists. J Jpn Soc Int Med. 2008;97:205–211. [in Japanese]. 10.2169/naika.97.205 18363217

